# Determination of dielectric properties of plaster blocks for sealing masonry using non-destructive frequency scanning methods

**DOI:** 10.1371/journal.pone.0295188

**Published:** 2023-12-07

**Authors:** Maria Elizabeth Teixeira Santana Praxedes, José Garibaldi Duarte Júnior, Erica Natasche de Medeiros Gurgel Pinto, Valdemir Praxedes Silva Neto, Kleber Cavalcanti Cabral, Adaildo Gomes d’Assunção

**Affiliations:** 1 Graduate Program in Civil and Environmental Engineering, Federal University of Rio Grande do Norte, Natal, RN, Brazil; 2 Department of Communication Engineering, Federal University of Rio Grande do Norte, Natal, RN, Brazil; Bahria University, PAKISTAN

## Abstract

With the increasing use of traditional and new models of wireless communication systems, the study and determination of the electrical characteristics of materials used in civil construction is an important topic to establish an understanding of how the radio frequency signal behaves inside built environments. This study presents an extensive process of characterization of electrical parameters of plaster blocks used in the construction of walls. Different from the literature where a prior estimation of the data occurs to enable sampling parameters to be obtained, this work proposes an innovative way of obtaining them entirely based on the analysis of the material through frequency measurements, which results in a greater level of precision of the data results. Analyses are made in the frequency range from 0.7 to 5.2 GHz, which has been used for several wireless communication standards. To carry out the electrical characterization of dielectric materials, a non-invasive methodology is proposed based on an innovative combination of the Nicolson–Ross–Weir Method (NRW) and the Ray Tracing Method. Through the proposed methodology and an extensive campaign of measurements using frequency scanning equipment, calculated and experimental data of Shielding Effectiveness (SE), complex relative electrical permittivity, loss tangent, attenuation coefficient and conductivity were obtained for a wide range of frequency, considering different samples of plaster blocks. The obtained results are compared to those available in the related literature, confirming the accuracy of the proposed analysis.

## 1. Introduction

Current and new generations of wireless communication, such as 5G and 6G, are imposing a series of critical conditions related to the electrical properties of the materials that constitute the environments through which electromagnetic waves propagate. In indoor environments, such as classrooms, offices, auditoriums and living rooms, the level of radiation from Wi-Fi, Bluetooth, radio and mobile phone services is high [1, 2]. Knowing how the constructive materials of these environments relate to the electromagnetic (EM) communication signal, in terms of attenuation, conductivity and dielectric characteristics, is extremely important and enables optimization performance under certain operating conditions. The characteristics of the materials commonly used in civil construction are preponderantly active in influencing the propagation of the EM wave [3]. Constructive specifications of physical dimensioning, chemical and molecular composition, porosity, water/humidity concentration and presence of internal metallic structures, can destructively affect the information signal of the main services currently used [4]. Therefore, it is essential to investigate and measure the electrical properties of these materials. Studies related to the modeling of losses in the propagation of EM waves in different media are usually based on the results of the characterization of these materials, mainly in relation to their respective electrical parameters.

Recently, several studies have addressed the electrical characterization of building materials. Reference [5] proposes the characterization of the complex electrical permittivity of concrete blocks using a new planar microwave sensor. A study on electrical permittivity and attenuation level in solid ceramic bricks for different levels of porosity and humidity is proposed in [6]. Approaches considering different civil construction parameters such as environment class, chemical composition, molar concentration, moisture and porosity are presented in [7–12] for the study of dielectric characteristics. One of the key parameters in the radio channel modeling is the loss factor due to the EM wave propagation through physical obstacles. Studies with analysis of the EM loss caused by walls in civil constructions are discussed and dimensioned in [3, 13–18]. Analysis methodologies are used based on the transmitted and received power ratio in antennas arranged in a specific setup.

In general, non-destructive methods, that is, methods that do not need to cause any kind of physical damage to the material under test (MUT), stand out among electromagnetic characterization applications. In [19] a classical approach is used based on the tracing of wavefront rays that propagate through homogeneously modeled walls, as mentioned in [20]. A study is carried out for different angles of incidence of the EM wave on concrete walls at 900 MHz. However, the method employed needs electrical permittivity values from the literature to obtain its final results. Based on the scattering parameters of systems with one and two ports, in [21, 22], the Nicholson-Ross-Weir (NRW) method is presented and used for characterizing materials, obtaining real and imaginary electrical permittivity characteristics and loss tangent. This method is also applied to characterize different materials, such as biological materials [23, 24].

Gypsum is a construction material that, due to interesting physical and mechanical properties, has been shown to be an excellent alternative for use in vertical sealing masonry. It is a material obtained through the calcination process of gypsum, in which the bihydrated calcium sulfate loses one and a half molecules of water, transforming it into semihydrated calcium sulfate [25, 26]. Depending on the speed of calcination, gypsum decomposition can result in gypsum with large, regular crystals, or gypsum with small, irregular crystals. When technically well built, gypsum block masonry has advantages such as good mechanical strength [27], greater thermal insulation [28, 29] and acoustic insulation [30], because of its low density, it imposes less effort on structural elements [27] and greater productivity and lower total cost for sealing. In addition, gypsum is produced with low energy consumption and is therefore ecologically correct.

The electromagnetic (EM) wave of a wireless communication service that propagates in different environments, such as civil constructions, will present power losses based on the materials that make up the environment in which it propagates. In view of this, it is essential to electrically characterize these materials in order to make possible the proposal of wireless communication systems sufficiently sized for operation in these passive attenuation environments. This work aims to present a new non-invasive methodology for the electrical characterization of solid materials used in civil construction. To validate the method, the process of determining the electrical parameters of plaster blocks used in the construction of walls is investigated. Two classic methods are combined and used in an innovative methodology: the NRW Method and the Ray Tracing Method. According to the literature, the Ray Tracing method usually uses permittivity values (real and imaginary) estimated and obtained from references. This fact can cause inaccuracy or low assertiveness in the results obtained for the sample under analysis. One of the points of innovation proposed in this work is precisely the use of the NRW Method as a way of obtaining the sample’s permittivity parameter, and with this data, obtaining other parameters with the Ray Tracing method, thus resulting in a higher level of assertiveness of results, since no data was estimated but obtained directly from the sample under analysis. The study and analysis of electrical parameters of Shielding Effectiveness (SE), complex relative permittivity, loss tangent, attenuation coefficient and conductivity considering different samples of plaster blocks, and for a wide range of frequencies (0.7 to 5.2 GHz) in which several wireless communication standards are inserted, are carried out. A measuring setup is described and used in an extensive measurement campaign on plaster blocks. The text is organized as follows: section 2 presents a discussion of the classical methodologies used and the proposed new method, as well as a description of the plaster block samples used; in section 3 the results obtained through measurements and calculated according to the proposed methodology are described, both being compared with values reported in the specialized literature; Finally, a conclusion is presented in section 4.

## 2. Materials and methods

In order to promote a concise presentation of the process of electrical characterization of plaster blocks used in the construction of walls, this section addresses the two methods used in this work. A logical scheme is described that allows the use of both techniques to, from data measured through a setup, obtain the electrical characterization of the blocks for a given frequency range. Also presented are the assembly characteristics of the measurement setup in radio frequency (RF) used to obtain the data. The constructive characteristics of the gypsum block samples used for characterization are shown.

### 2.1 NRW method

**Fig 1:**
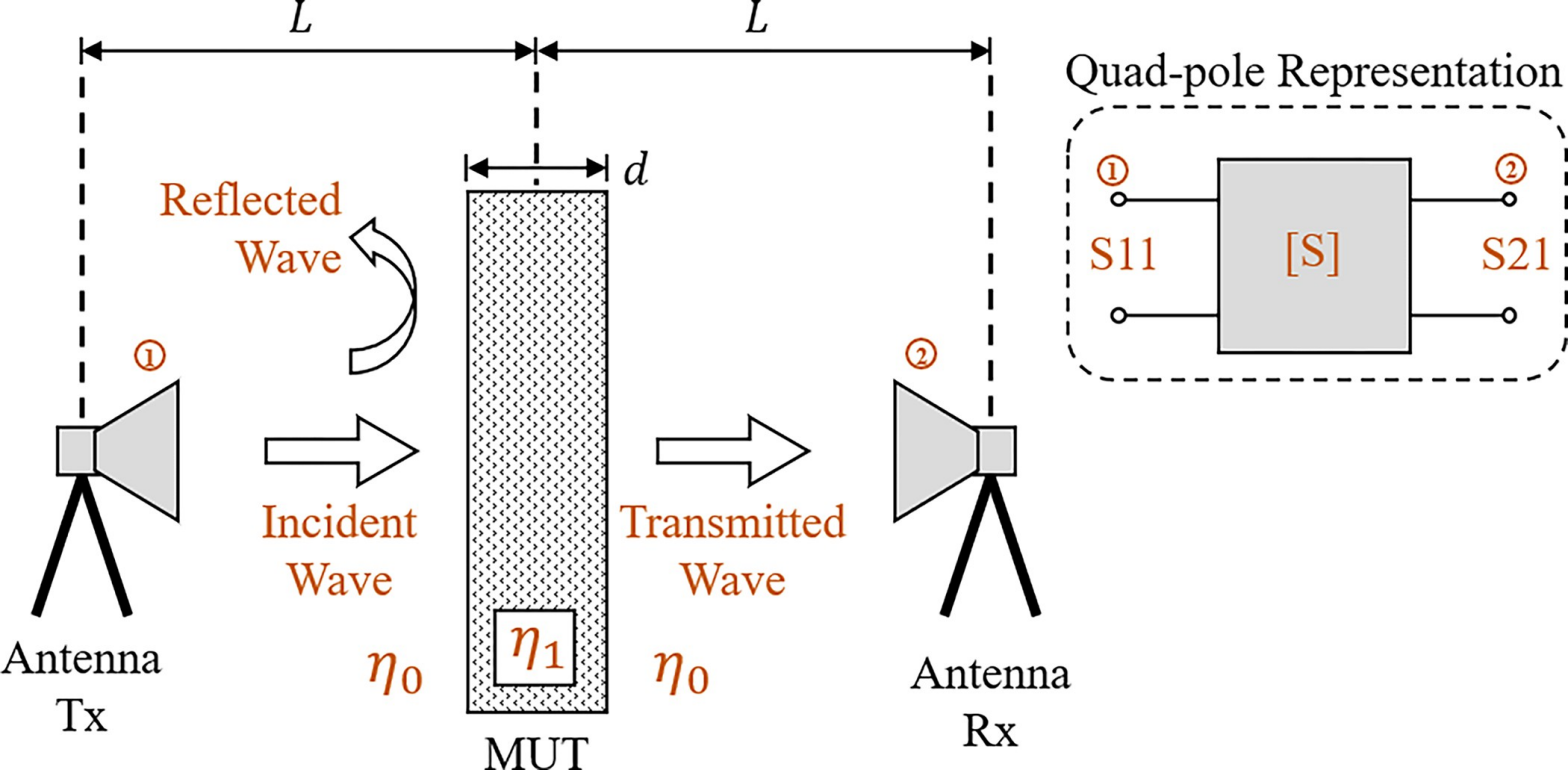
Measurement scheme to obtain [S] parameters using Tx, Rx antennas and material under test (MUT).

One of the most used methods in the characterization of electrical permittivity is the Nicolson–Ross–Weir (NRW) [5, 21, 22]. Using relatively simple equations, the method allows obtaining complex relative permittivity parameters $\varepsilon_{r}=\varepsilon_{r}^{\prime }-j\varepsilon_{r}^{\prime \prime }$ and complex relative permeability *μ*_*r*_. From obtaining the scattering parameters [S] of reflection and transmission as a function of frequency, as shown in Fig 1, and data on sample thickness and distance between transmitter/sample/receiver, it is possible to obtain the electric permittivity of the material under test (MUT) for a given frequency range.

Considering a plaster block used in the construction of walls as a homogeneous sample, the transmitting device (Tx-antenna) emits an electromagnetic wave at a certain frequency. Then, an incident plane wave hits the plaster block surface. At this time, two phenomena occur. A reflected wave is generated from the impedance mismatch *η* at the interface between the external medium (free-space/air—*η*_0_) and the internal medium of the MUT (gypsum block— *η*_1_). This reflected wave can be represented in terms of parameters [S], with S11 being the factor that relates the incident wave and the reflected wave. The second phenomenon is the transmission of a wave through the drywall, which is captured by the receiving device (antenna-Rx). This wave, represented in terms of parameters [S], is identified as S21, which relates the incident wave and the transmitted wave. Both parameters S11 and S21 have in essence the influence of the electrical properties of the gypsum block under test. S11 considers the reflection due to the impedance mismatch at the air-gypsum interface, and S21 considers the losses generated by the transmission of the electromagnetic wave through the plaster block.

According to the NRW method [21], the reflection coefficients *Γ* and transmission *T* are expressed by the scattering parameters in complex form as a function of the frequency *f*, as:

$$\Gamma =K\pm \sqrt{K^{2}-1}$$ (1)

$$T=\frac{S11+S21+\Gamma }{1-(S11+S21)\Gamma }$$ (2)

with

$$K=\frac{S11^{2}-S21^{2}+1}{2S11}$$ (3)

The sign value in (1) is chosen so that the condition |*Γ*|≤1 is true. In the application of this method, the propagation channel that includes the gypsum block under test is modeled as a transmission line, that is, a network of two ports (also known as a quadripole) and has its frequency response represented by its scattering parameters [S] [5]. Considering that gypsum fits the condition of non-magnetic material (*μ*_*r*_ = 1), the complex relative permittivity for the structure under test can be written as:

$$\varepsilon_{r}={\left[(\frac{jc}{\omega L})\ln T\right]}^{2}$$ (4)

Where *c* represents the speed of light in free space (3.0×10^8^ *m*/*s*), *ω* is the angular frequency (*ω* = 2*πf*) and *L* is the distance between Tx, the plaster block and Rx. Therefore, it is possible, through the given scattering parameters, the physical dimensions of the measurement setup and the thickness of the block, to obtain the characterization of the relative electrical permittivity, which is composed of the real component $\varepsilon_{r}^{\prime }$ and the imaginary component $\varepsilon_{r}^{\prime \prime }$. In addition, another parameter can be obtained from the real (*Re*) and imaginary (*Im*) components of *ε*_*r*_. It is the electrical loss tangent tan*δ*, given by *Im*(*ε*_*r*_)/*Re*(*ε*_*r*_).

### 2.2 Ray tracing method in walls

**Fig 2:**
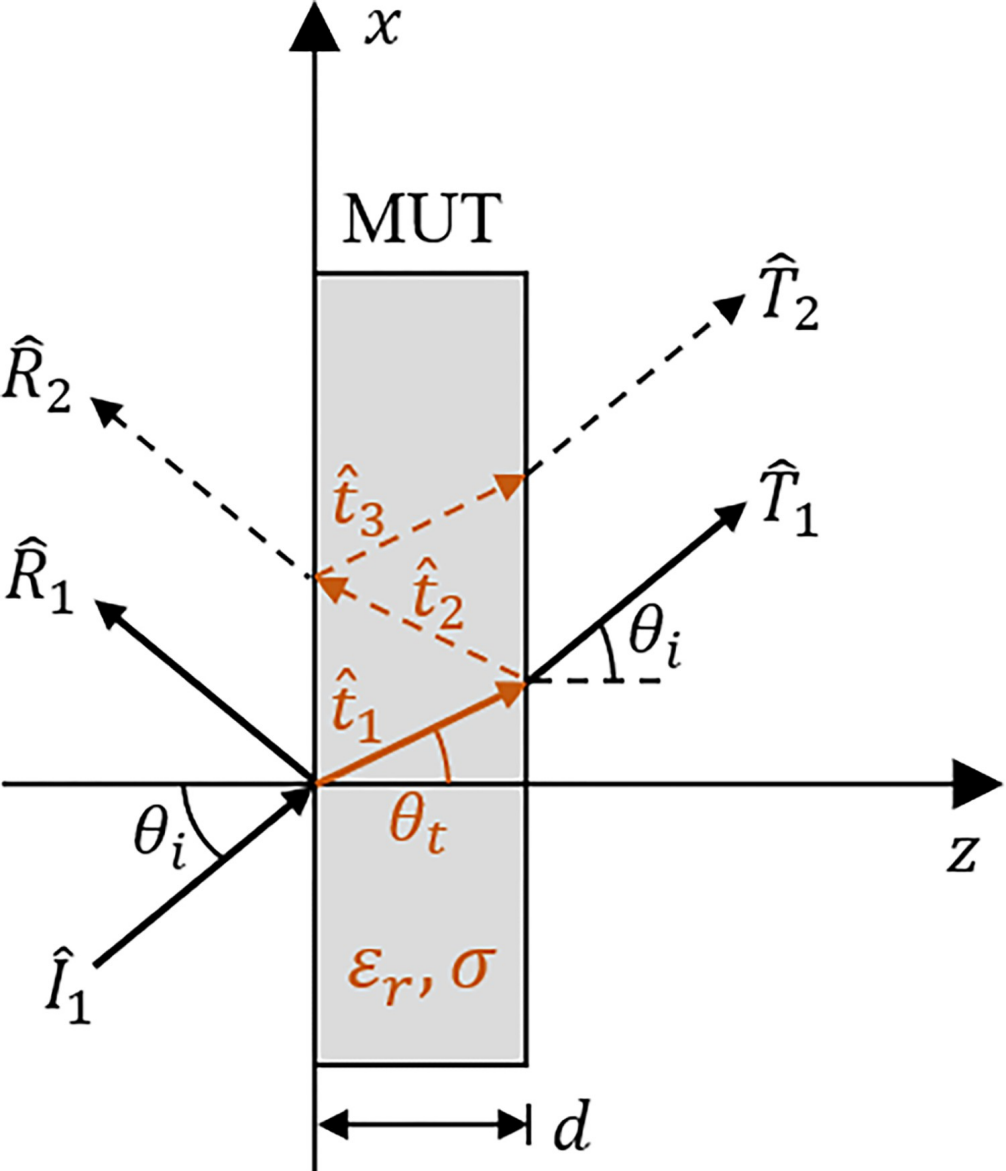
Ray tracing method. Illustration for single and multiple rays.

In the study of the propagation of EM waves and their interaction with obstacles, a wall can be considered as an almost infinite homogeneous flat block represented electrically by the parameters of relative permittivity *ε*_*r*_ = *ε*/*ε*_0_, conductivity *σ* and thickness *d*. In [19], a classical approach is presented in which a plane wave hits the surface of an almost infinite wall. In this situation, two phenomena are observed: the reflection of the EM wave and its refraction towards the medium inside the wall, a second refraction also occurs towards the medium outside the wall. That is, the reflection of the EM wave on the surface of the wall and transmission of the wave through the structure of the wall is verified. Fig 2 shows the schematization of the incidence, reflection, refraction and transmission ray tracings of the plane wavefront, given a wall with the following main parameters: electric permittivity, *ε*_*r*_, conductivity, *σ*, and thickness, *d*. Using the ray tracing method, there are two ways to approach these phenomena:

- Single ray model–the incident wave described by ${\hat{I}}_{1}$ hits the surface of the wall obliquely. It generates a reflected wave described by ${\hat{R}}_{1}$ and a refracted wave ${\hat{T}}_{1}$ inside the wall. A transmitted wave *T*_1_ is then observed outside the wall, as shown in Fig 2;
- Multiple-ray model–similar to the single-ray model, but the phenomenon of multiple refractions inside the wall is considered, thus resulting in multiple reflected and transmitted wavefronts (${\hat{T}}_{1},{\hat{T}}_{2}$ and ${\hat{T}}_{3}$), as described in Fig 2.

Considering the classical approach [31], for an interface between low-loss media, the incidence of a uniform plane wave on this interface results in a uniform plane wave reflected and another transmitted. However, in the case of quasi-conductor homogeneous media or those with considerable losses, as is the case with walls used in civil construction, the transmitted wave is converted into a non-uniform model. For this non-uniformity condition, the propagation constant *γ* = *α*+*jβ* is modified to *γ*_*m*_ = *α*_*m*_+*jβ*_*m*_, being (*α*, *α*_*m*_) and (*β*, *β*_*m*_) the corresponding attenuation and phase constants, respectively. These parameters can be written according to [19, 29]:

$$\gamma_{m}=\alpha_{m}+j\beta_{m}\to \left(\begin{array}{c} \alpha_{m}\\ \beta_{m} \end{array}\right)=\sqrt{\frac{1}{2}\{\left(\begin{array}{c} Re(\gamma^{2})\\ -Re(\gamma^{2}) \end{array})+\beta_{0}^{2}\sin^{2}\theta_{i}+|\beta_{0}^{2}\sin^{2}\theta_{i}-\gamma^{2}\right|\}}$$ (5)

with

$$\begin{array}{c} \gamma =\alpha +j\beta \\ \left(\begin{array}{c} \alpha \\ \beta \end{array}\right)=\omega \sqrt{\mu_{0}\varepsilon_{0}\varepsilon_{r}}∙\sqrt{\frac{1}{2}\{(\begin{array}{c} -1\\ 1 \end{array})+\sqrt{1+{\left(\frac{\sigma }{\omega \varepsilon_{0}\varepsilon_{r}}\right)}^{2}}\}} \end{array}$$ (6)

Where $\beta_{0}=\omega \sqrt{\mu_{0}\varepsilon_{0}}$ is the wave number in free space.

From ray tracing theory [19], the sum of rays transmitted through the homogeneous wall can be expressed as the amplitude of the transmission coefficient $\tilde{T}$:

$$\tilde{T}=T_{12}A$$ (7)

$$A=e^{-\alpha t}$$ (8)

Where *T*_12_ represents the Fresnel transmission coefficient for different incidence angle conditions *θ*_*i*_ [20]. For the condition of incidence of a vertically polarized plane wave on the surface of the structure (*z* = 0), *T*_12_ can be expressed according to (9). The *A* term corresponds to the amplitude attenuation factor due exclusively to the losses generated by the internal absorptions along the path *t* inside the wall, with *α* being the attenuation coefficient highlighted in (5) and (6).

$$T_{12}=\left|\frac{4\;\cos\theta_{i}\sqrt{\varepsilon_{r}-\sin^{2}\theta_{i}}}{{\left(\cos\theta_{i}+\sqrt{\varepsilon_{r}-\sin^{2}\theta_{i}}\right)}^{2}}\right|$$ (9)

Given this modeling, it is possible to establish a series of equations that relate two key parameters of the electrical characterization of walls, such as the relative permittivity *ε*_*r*_ and the conductivity *σ*. Considering a numerical study and obtaining attenuation data in frequency sweep measurements for different incidence angles *θ*_*i*_ on test walls of thickness *d*, it is possible to accurately determine such parameters as discussed in [19].

### 2.3 Electrical characterization of plaster blocks

To determine the electrical characteristics of plaster blocks used in the construction of walls, it is proposed a combination of two analysis methodologies, the NRW method and the ray tracing method, both discussed in the previous subsections. Unlike approaches available in the literature, such as [20], in which parameters are inferred or estimated to make it possible to obtain others. In the proposed approach all the electrical parameters that characterize the plaster blocks are obtained directly through experimental measurements or through processing the data obtained in the measurements. In this way, it is possible to characterize the MUT without the need to infer, estimate or carry out bibliographic surveys in the literature, mitigating the margin of error of the characterization results.

**Fig 3:**
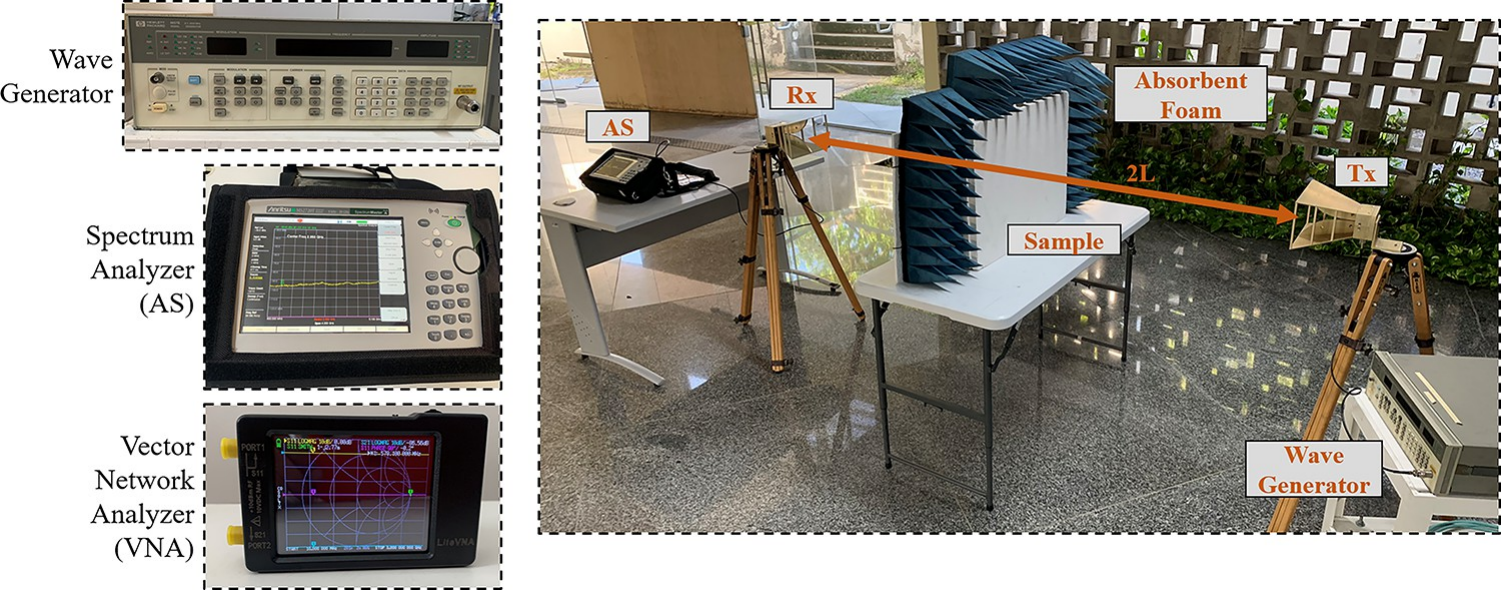
Setup and equipment assembled for frequency sweep measurements.

Fig 3 presents the setup used to carry out the measurements of the samples of plaster blocks. To obtain measurement data of S parameters and attenuation, two analyzers, commonly used in RF experiments, are used: Vector Network Analyzer (VNA) and Spectrum Analyzer (SA). In the process of measuring the attenuation levels, a Wave Generator is used. The VNA used was the LiteVNA model from the manufacturer mRS. The SA with model MS2720T from Anritsu. A Hewlett Packard (HP) model 8657B generator was used. A set of commercial antennas, model SAS-571 of A. H. Systems, (Tx and Rx) of the horn type with a directive radiation diagram, operating frequency between 0.7 and 18 GHz, and variable gain between 1.4 and 15 dBi is used. The antennas are arranged at a distance *L* from the MUT and it is considered the condition in which an antenna is positioned in the far field region of each other. According to this condition, the expression 2*L*≥2*D*^2^/*λ* must be satisfied, where *D* is the largest dimension of the antenna (equal to 24.0 cm) and *λ*, the wavelength of the RF signal. For this, 2*L* equal to 2.0 m was used. Measurements are carried out in an external environment in order to mitigate the effects of reflection and multipath transmission, which can cause inaccuracy in the RF measurement data. Furthermore, absorbing foams are used around the MUT samples, in order to reduce the previously mentioned effects and preserve the RF signal that enters the MUT sample and reaches the Rx-antenna.

**Fig 4:**
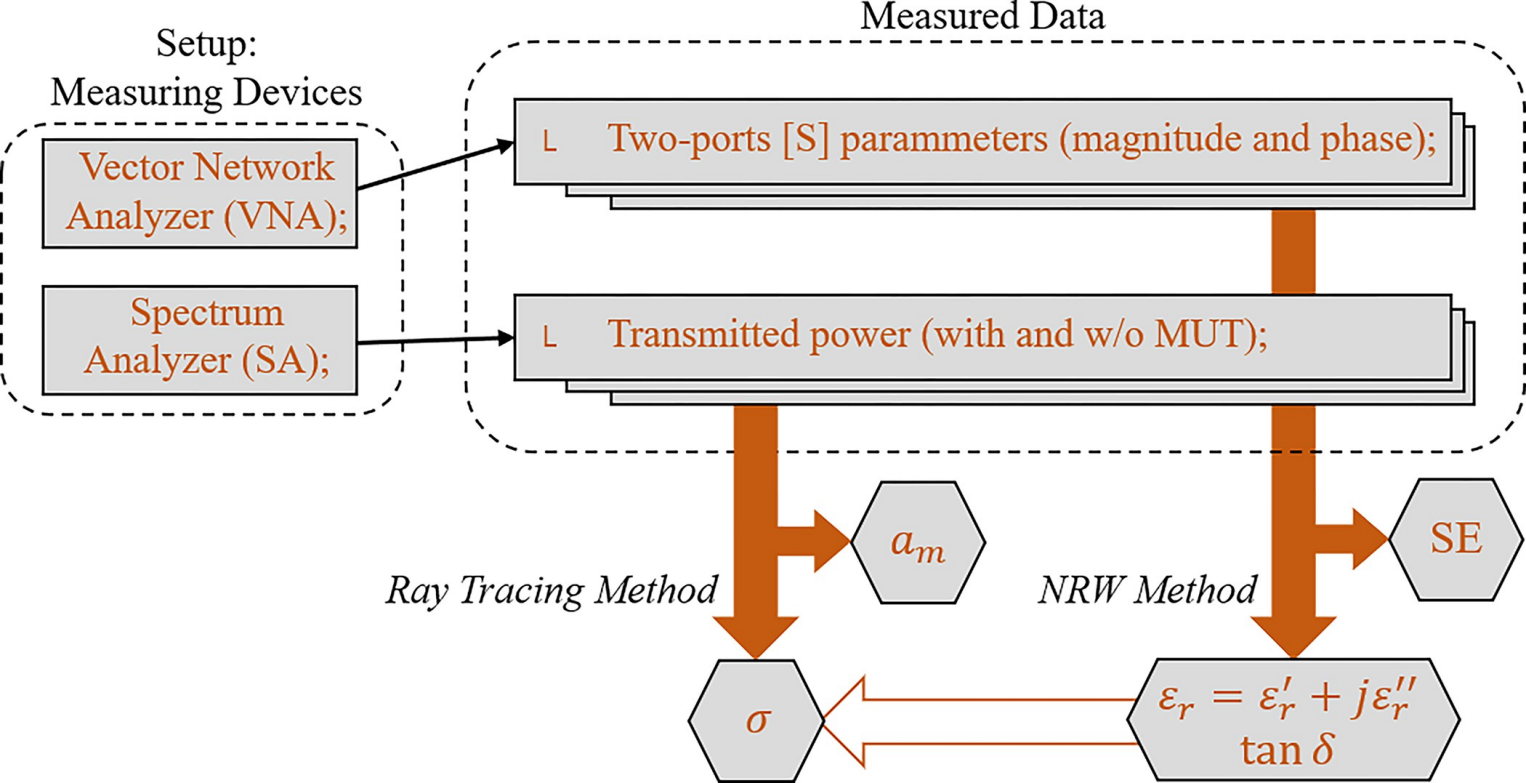
Flowchart for the electrical characterization of plaster blocks.

From the data of scattering parameters [S] and power received at the Rx-antenna, obtained through the Vector Network Analyzer (VNA) and the Spectrum Analyzer (AS), respectively, the information is recorded and subjected to a series of analyses, which result in the electrical parameters of the gypsum block subjected to measurement. In Fig 4, the steps are highlighted in a sequential and schematic way that summarizes the methodology used to characterize the gypsum block samples. In both measurement devices, a frequency sweep is performed to obtain the measured data. In the following sections, analyses are carried out considering the entire measurement range. However, ten frequencies were chosen between 0.9 and 5.0 GHz belonging to wireless communication standards commonly used in built environments are highlighted, including mobile telephony of different generations (3G, 4G and 5G), Wi-Fi and Bluetooth, presented in Table 1. As shown in Fig 4, the VNA is responsible for registering the values of the [S] parameters in complex form (magnitude and phase) as a function of frequency for the two ports (port1: antenna-Tx, port2: antenna-Rx). The AS performs the measurement of the RF power captured in the Rx-antenna from the incident wave coming from the Tx-antenna connected to the Wave Generator. These measurements are performed for each considered frequency.

**Table 1:** Wireless communication standards and operating frequencies.

Frequency of Operation (GHz)	Wireless Communication Standard	Reference
0.90	Mobile telephony and Wi-fi (IEEE 802.11ah)	[32]
1.50	Mobile telephony (3GPP)	[33]
1.80	Mobile telephony (previous generations and 5G)	[33]
2.10	Mobile telephony (3GPP)	[33]
2.30	5G Technology Standard	[34]
2.45	Wi-fi (IEEE 802.11ah)	[35]
3.30	Mobile telephony (5G)	[34]
3.60	Wi-fi (IEEE 802.11y)	[36]
3.70	Mobile telephony (5G)	[34]
5.0	Wi-fi (IEEE 802.11a/h/j/n)	[32]

In the measurements campaign, the situation without MUT sample is initially considered, which is the situation of incidence of the EM wave in free space and is then taken as a reference for the other measurements and analyses. The data obtained from the measurements of [S] are used to initially obtain the Shielding Effectiveness (SE) parameter that demonstrates the level of attenuation caused by the MUT in the link of the considered two-port system. Eq 10 presents the expression for SE in dB, where *S*21_0_ is the free-space transmission coefficient (without MUT sample), and *S*21_*MUT*_ is the transmission coefficient for the situation with MUT sample [18]. Then, the data obtained for the scattering parameters, [S], are used in the NRW Method to extract the values of the complex relative permittivity ε_r_ and the loss tangent. With the transmitted power data between the Tx and Rx antenna for different operating frequencies and considering four incidence angles *θ*_*i*_ between 0° and 45°, the measured attenuation coefficient parameter *a*_*m*_ in dB is initially computed, obtained directly from the difference between the power received at Rx in the reference case (without MUT) and with the MUT, according to Eq 11. From the permittivity values obtained via the NRW Method, the conductivity value for the MUT is obtained considering Eqs (5–9), thus concluding the electrical characterization of the gypsum block used in the construction of walls.


$$SE=S21_{0}-S21_{MUT}$$ (10)

$$a_{m}={P_{Rx}}_{0}-{P_{Rx}}_{MUT}$$ (11)

### 2.4 Description of the used plaster blocks

**Fig 5:**
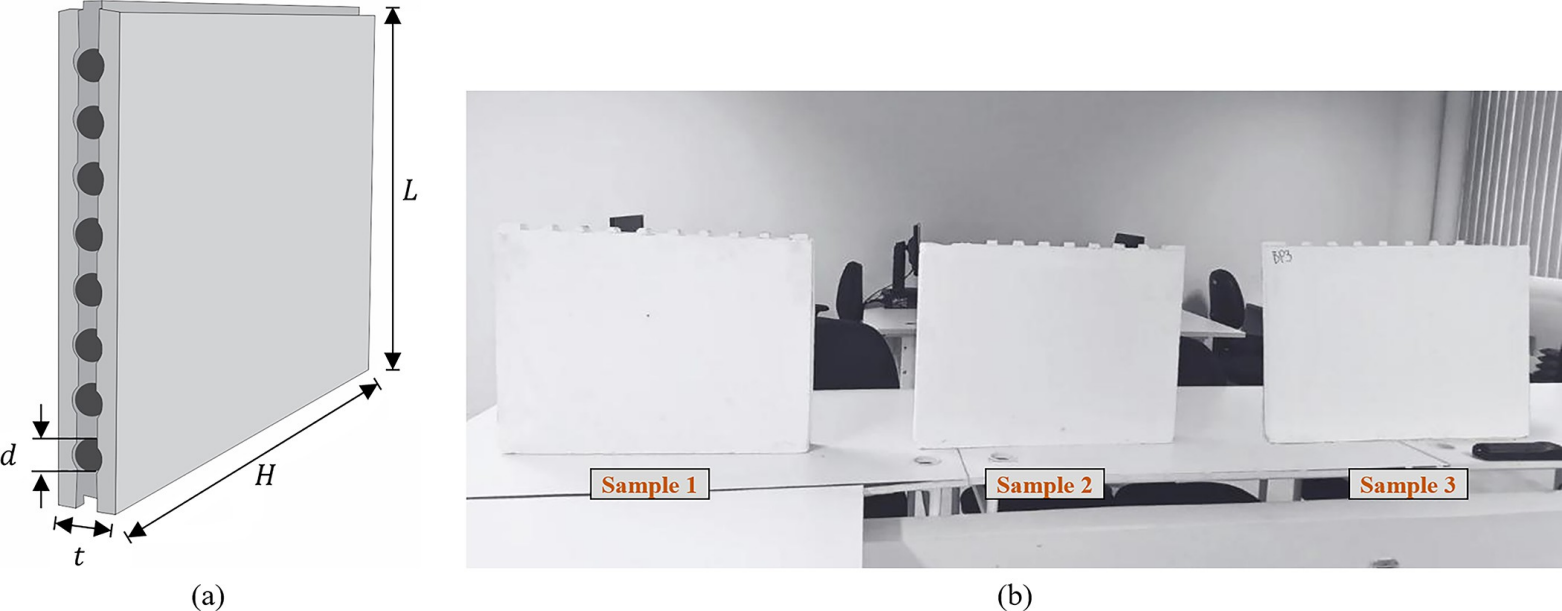
Samples of plaster blocks. (a) Block Profile. (b) Considered Samples.

In order to determine the dielectric properties of plaster walls and evaluate the effect of this type of masonry on the propagation of electromagnetic waves, three samples of conventional white plaster blocks for masonry from the same lot are analyzed. The samples considered were manufactured industrially, with a parallelepiped shape, cylindrical holes and two flat and smooth faces, as shown in Fig 5.

The chemical composition of the gypsum used to manufacture the samples was determined using an X-ray fluorescence spectrometry (XRF). The Chemical analysis result of gypsum plaster is presented in Table 2. Result shows that the tested gypsum plaster is too rich in calcium oxide and in sulfuric anhydride.

**Table 2:** Chemical composition (% by mass) of gypsum in oxides.

Component	(%)
SO_3_	53.269
CaO	45.767
SiO_2_	0.810
K_2_O	0.154

Considering the results in Table 2, the CaO content meets the requirements of the ABNT NBR 13207/2011 standard, which determines a minimum content of 38%, given that the analysis obtained 45.767% for this component. The SO_3_ content also meets the range specified by the same standard (minimum of 53%), resulting in a percentage of 53.269%.

The physical properties of each of the samples shown in Fig 5(B) were determined following the recommendations and procedures established by NBR 16495/2016, NBR16494/2017 and ASTM C1396. According to NBR16.494/2017, the physical dimensions are determined by the thickness t, length L and height H. The preferred thicknesses for plaster blocks used in masonry are 50 mm, 70 mm, 76 mm, 80 mm and 100 mm with a tolerance of + 0.5 mm; the length must be (666.00 + 3.0 mm) and the height (500.00 + 0.5) mm. The physical dimensions for the analyzed samples are shown in Table 3.

**Table 3:** Physical dimensions of the samples determined according to NBR 16.495/2016.

Parameter (mm)	Sample 1	Sample 2	Sample 3
*L*	667.97	668.53	667.97
*H*	500.00	500.00	500.00
*t*	76.20	76.26	76.27

According to the values presented in Table 3, the samples of plaster blocks analyzed in this work meet the requirements specified in the standard related to physical dimensions, so that the plaster blocks can be applied in the execution of masonry. In addition to the physical dimensions, it was verified whether the samples have an apparent density *ρ* that meets the normative specifications of 800.00 Kg/m^3^ < *ρ* < 1100.00 Kg/m^3^. The values obtained for the sample densities were *ρ*1 = 890.69 kg/m^3^, *ρ*2 = 948.99 kg/m^3^ and *ρ*3 = 919.64 kg/m^3^, with an average apparent density of *ρ*_*avg*_ = 919,77 kg/m^3^ and average standard deviation equal to 3.17%. Thus, it can be stated that all samples meet the normative specifications for the density criterion, and can be characterized as medium-density gypsum blocks.

All blocks considered were stored in a dry and protected place, guaranteeing a humidity lower than 4%. In addition, none of the samples had fillings, cracks, breaks or stains that could make the final finish of the wall unfeasible.

## 3. Measurements and experimental results

### 3.1 Characterization of complex relative electrical permittivity

**Fig 6:**
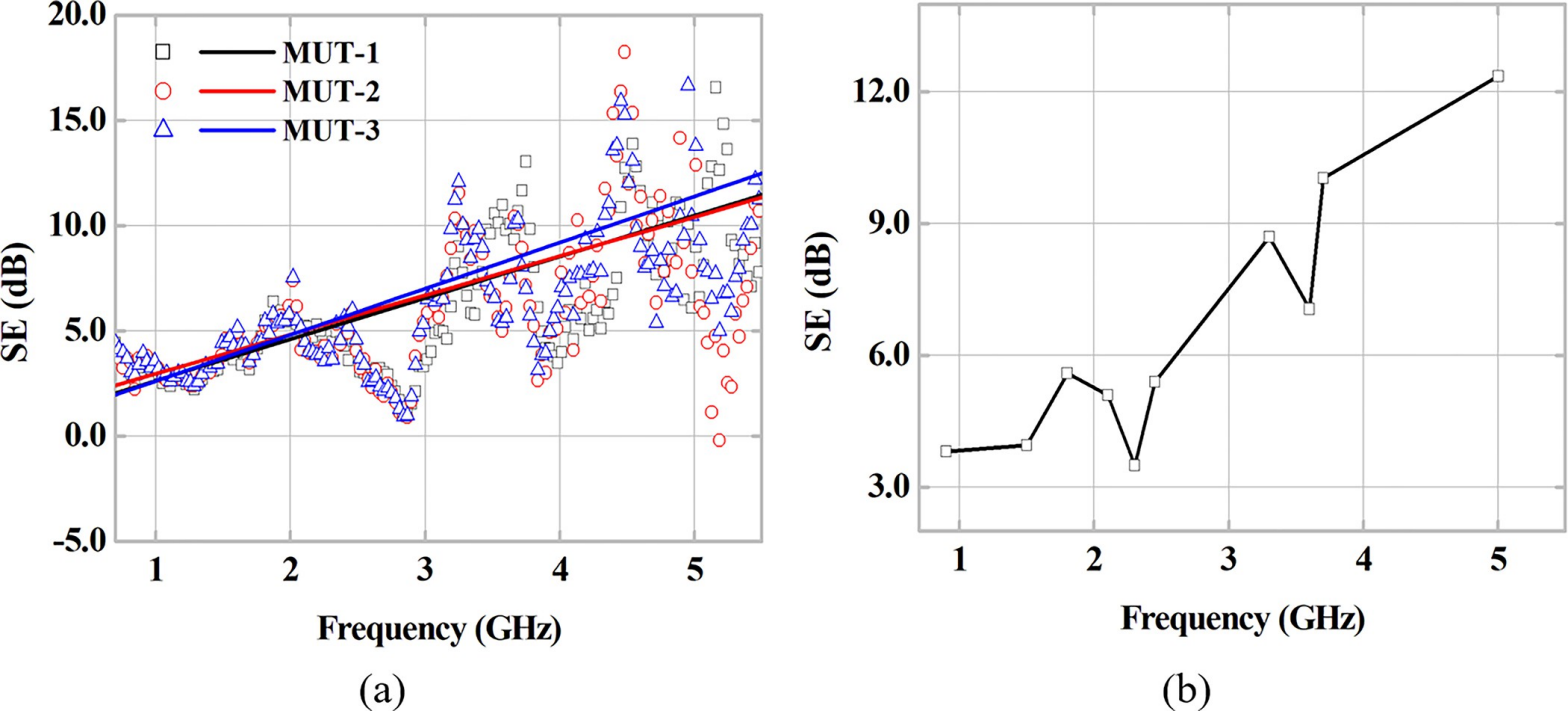
SE parameter as a function of frequency. (a) Obtained results and fitting for three different MUT samples. (b) Mean SE results for frequencies of interest.

Following the chronology presented in the previous section, we initially discuss the experimental results derived from the measurements of the scattering parameters [S] from the VNA for each of the three MUT samples, according to ambient temperature and pressure conditions. The reference case (without MUT sample) and with MUT sample for a frequency measurement range from 0.7 to 5.5 GHz was considered. In this first moment, the measurements were carried out taking the case of normal incidence of the plane wave on the MUT surface, that is, a *θ*_*i*_ = 0°. Fig 6 shows the measured points of the Shielding Effectiveness (SE) parameter. Despite the observed dispersions, the regression lines plotted for each MUT sample demonstrate the behavior of SE increase as a function of the measurement frequency. This type of frequency response can be considered valid, since according to the literature [18], the increase in frequency in the microwave range causes a natural increase in losses in the RF signal. These losses can be estimated through the SE parameter, according to its definition. Fig 6(B) highlights the average SE in relation to the different MUT samples (1–3) for the frequencies of interest shown in Table 1. The lowest value was observed at 2.3 GHz with SE of 3.49 dB, the highest value of SE of 12.36 dB was verified at the frequency of 5.0 GHz, used in different Wi-Fi standards.

**Fig 7:**
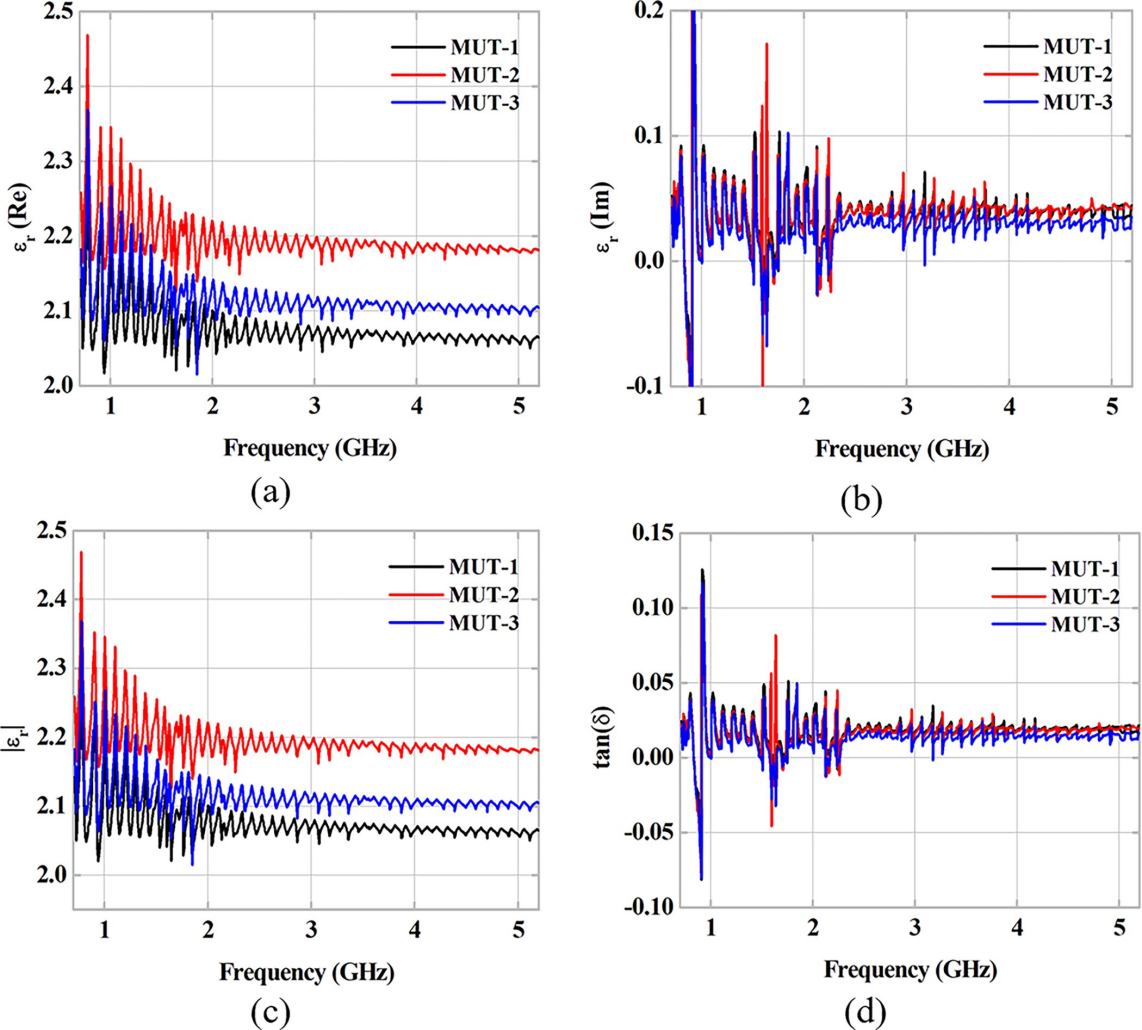
Data obtained via NRW method. Relative Electrical Permittivity: (a) Real and (b) Imaginary Parts. (c) Module. (d) Dielectric loss tangent.

Applying the NRW Method, described in subsection 2.1, with the obtained scattering parameters [S], of the measuring campaign and with the dimensions of three different MUT samples, the values of complex permittivity *ε*_*r*_ and tangent of electrical losses tan*δ* are obtained. The results of the method are shown in Fig 7. In the analyzed initial frequencies, a dispersion of 0.5 is observed. However, the $\varepsilon_{r}^{\prime }$ and $\varepsilon_{r}^{\prime \prime }$ curves demonstrate a balance in the range of 2.04–2.2 and 0.023–0.042, respectively, along the measurements for the three MUTs. For the loss tangent curves, a similar behavior is observed, with an equilibrium level close to 0.017 being obtained. Table 4 presents a summary with the results obtained, mean and standard deviation, in addition to a comparison with measurements available in the literature. Average values of 2.1284 and 0.0163 are obtained for the relative electrical permittivity value and loss tangent, respectively, for the plaster blocks used in the wall construction. Considering the structural composition containing cylindrical holes along the entire vertical extension of the samples (1–3), as described in subsection 2.4. The relative permittivity data obtained were slightly below the values found in the literature, which are obtained through massive samples of gypsum blocks. With a relative difference close to 20%, the presence of air-filled holes in the MUT may explain the decrease in relative electrical permittivity values when compared to those available in literature. Therefore, the obtained values can be considered consistent with the developed analyses, and, in turn, can be used in other analyses.

**Table 4:** Summary of the results obtained via the NRW method for the measured plaster blocks in 0.7–5.5 GHz frequency range.

	MUT-1	MUT-2	MUT-3		Average	Standard Deviation
$\varepsilon_{r}^{\prime }$	2.0751	2.1948	2.1150		**2.1283**	0.0609
$\varepsilon_{r}^{\prime \prime }$	0.0374	0.0176	0.0131		**0.0227**	0.0129
|*ε*_*r*_|	2.0754	2.1948	2.1150		**2.1284**	0.0608
tan *δ*	0.0181	0.0176	0.0131		**0.0163**	0.0028
**Reference**				**|*ε***_***r***_ **|**		
[37]				2.94		
[38]				2.73		
This Work				**2.1284**		

### 3.2 Characterization of the attenuation and conductivity coefficient

After the measurement of the scattering parameters, [S], the transmitted power by the Tx-antenna and the received power by the Rx-antenna are measured for a total of 44 frequencies over the range from 0.9 to 5 GHz. For this, a Wave Generator and a Spectrum Analyzer are used. Measurements are performed for two cases, without MUT sample and with MUT samples, for different angles of incidence *θ*_*i*_ of the plane waves on the MUT surface. Also, considering the standard width and length dimensions of plaster blocks (MUT), as discussed in subsection 2.4, an analysis is performed for different values of the MUT sample thickness and the corresponding effect on the attenuation level caused in the RF link.

**Fig 8:**
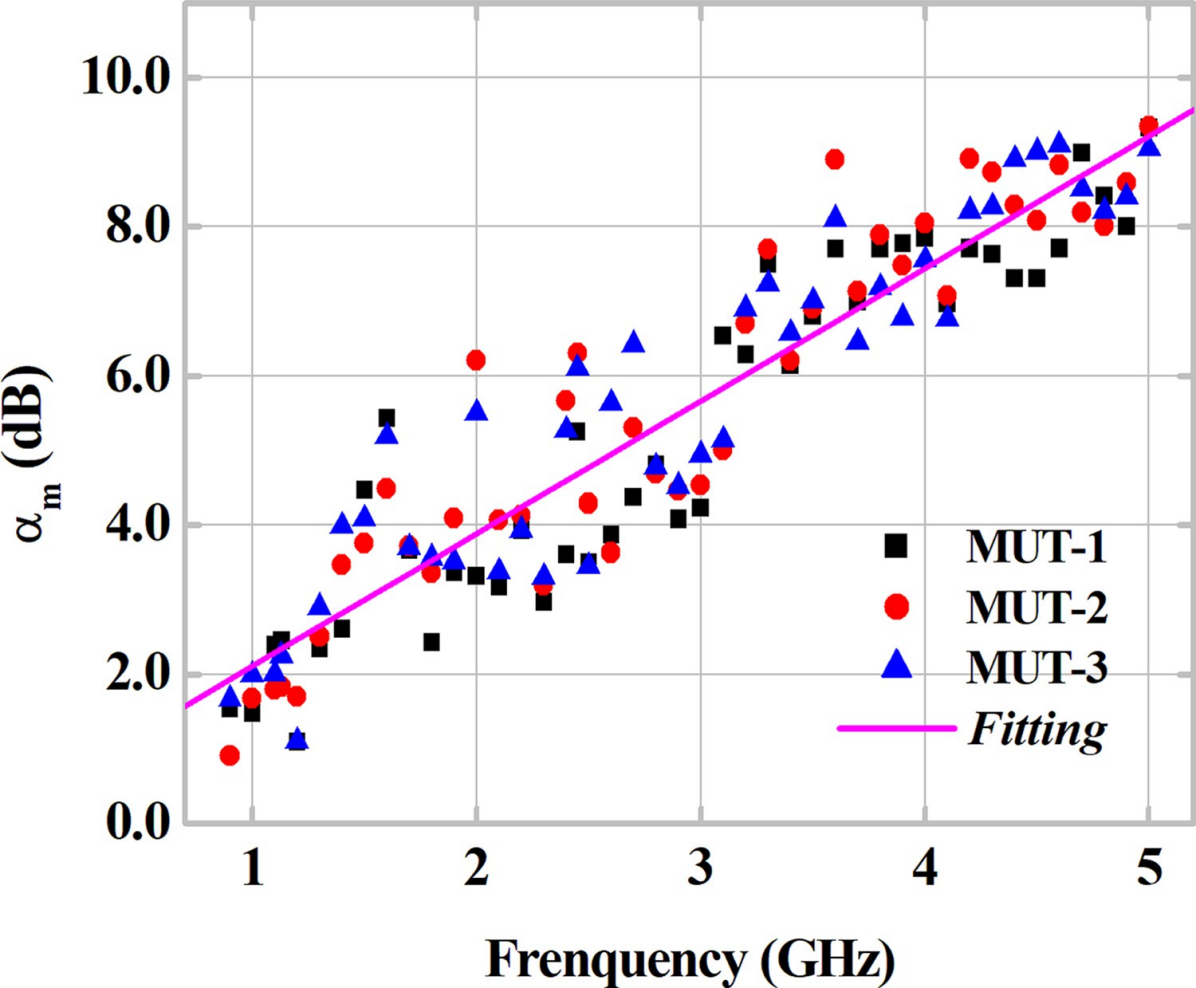
Measured attenuation coefficient results as a function of frequency for the three MUTs. Fitting result with midpoints.

Taking the measured attenuation coefficient *a*_*m*_(dB) as the direct difference between the power received in the reference condition, named as without MUT sample, and the power received with MUT sample, the obtained values for *a*_*m*_ for each analyzed MUT are shown in Fig 8. A minimum attenuation value of 0.91 dB is verified for MUT-2 at 0.9 GHz, with a maximum level of 9.34 dB at 5 GHz. Considering the dispersion on the measured attenuation coefficient *α*_*m*_ (dB) results shown in Fig 8, an Average Standard Deviation of approximately 0.46 is calculated. Considering the average fitting curve between the three MUTs, a measured attenuation trend of 1.77 dB/GHz is verified, with a correlation index (*R*^2^) of 94.859% with the distribution of sample points. In general, the points for the three samples show a trend towards an increase in attenuation as a function of an increase in the operating frequency of the RF signal, a behavior that is consistent with what was expected.

**Fig 9:**
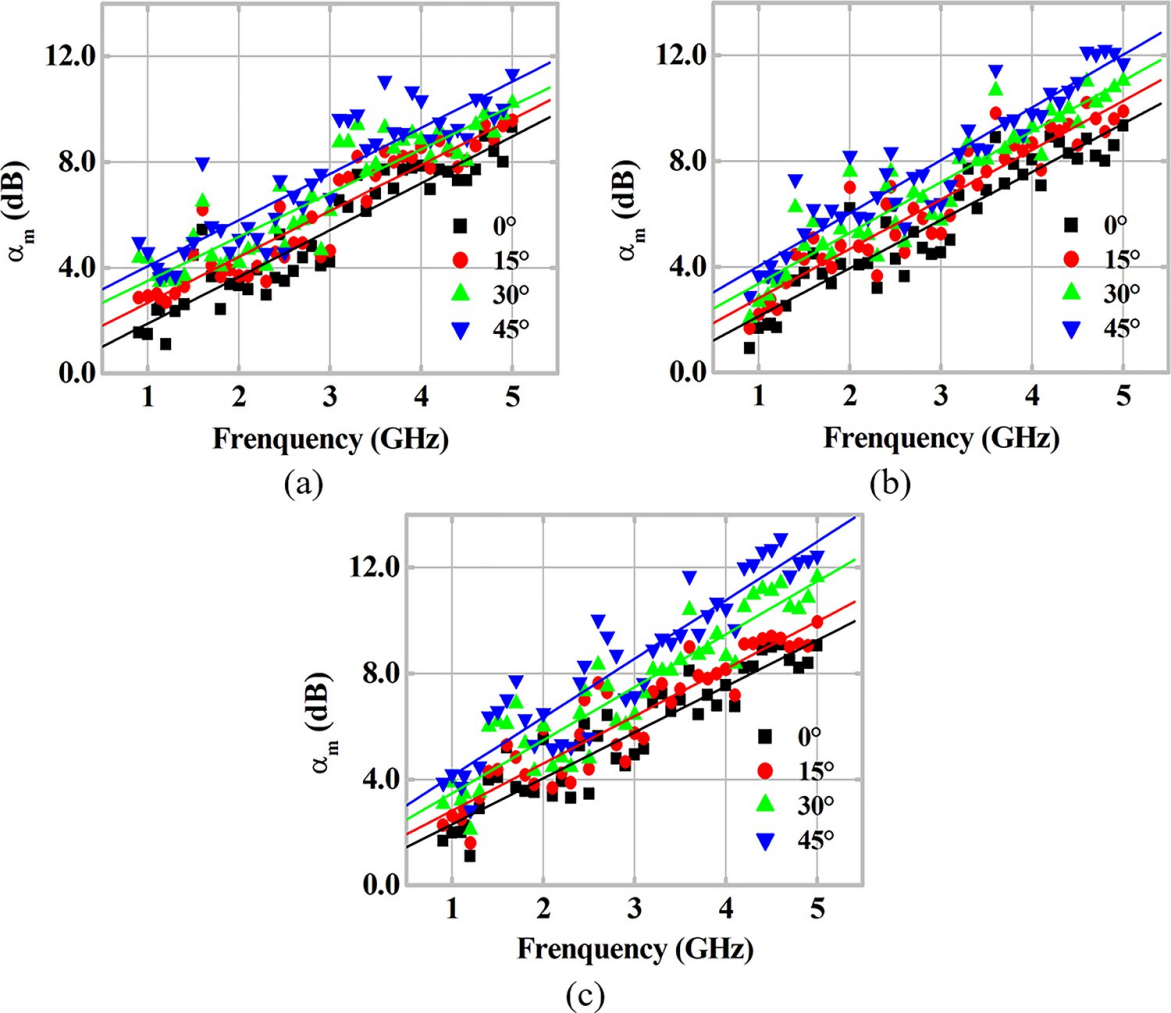
Analysis of the incidence angle *θ*_*i*_ on the measured attenuation coefficient *α*_*m*_ as a function of frequency for (a) MUT-1, (b) MUT-2 and (c) MUT-3.

In addition, the study of the incidence angle *θ*_*i*_ and its influence on the transmission coefficient for each MUT sample is carried out, and the obtained results are shown in Fig 9, for *θ*_*i*_ values between 0° and 45°. According to Snell’s Law, with respect to the wave transmission to the MUT sample, and the theory presented in subsection 2.2 [19, 39], the increase in *θ*_*i*_ results in a quantitative and qualitative increase in internal transmissions in the MUT and consequently in the losses caused by it. Therefore, for larger incidence angles, the power received at the Rx-antenna must be smaller, and a higher value of *α*_*m*_ is expected. This behavior is obtained at the points measured throughout the analysis for the three MUT samples. For the measured frequency range, both the increasing trend of *α*_*m*_ is observed for higher frequencies, as well as higher attenuation levels for higher *θ*_*i*_. The dispersions of the measured points demonstrate this trend, according to the fitting lines for each set of points highlighted in Fig 9.

**Fig 10:**
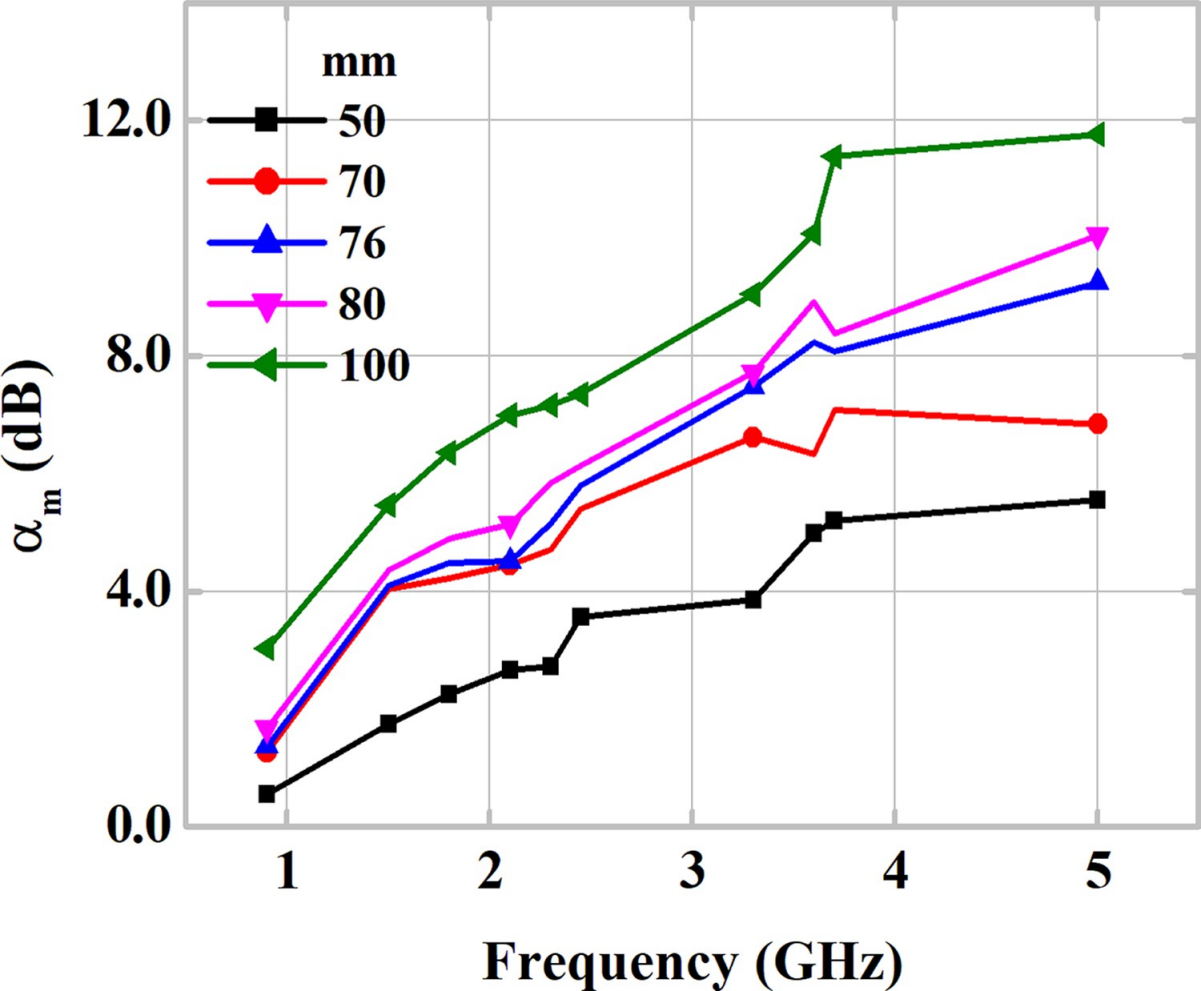
Analysis of the plaster block thickness on the measured attenuation coefficient *α*_*m*_ as function of frequency.

Also, the analysis of the *α*_*m*_ points is performed considering plaster block samples of different thicknesses (50, 70, 76, 80 and 100 mm) for the frequencies of interest (Table 1), as shown in Fig 10. The attenuation was measured in fifteen samples of plaster blocks, where three samples of each of thicknesses. All samples used meet the requirements established by standards. An increase in attenuation levels is verified considering greater thicknesses. For a sample with a minimum thickness of 50 mm, a minimum and maximum attenuation of 0.55 and 5.56 dB, respectively, is verified. As for the thicker sample, attenuations measured between 3.03 and 11.76 dB are verified. The increase in sample thickness represents an increase in the amount of lossy material, which in turn causes an increase in attenuation levels in the RF link.

Then the attenuation coefficient data measured as a function of frequency and of the incidence angle *θ*_*i*_, combined with the obtained relative electrical permittivity values in the complex form ($\varepsilon_{r}=\varepsilon_{r}^{\prime }+j\varepsilon_{r}^{\prime \prime }$), the investigation of the *σ* conductivity is carried out based on the Ray Tracing Method, discussed in subsection 2.3. Using Eqs (5–9), it is possible to derive a series of matrices as a function of frequency, with the modified transmission coefficient *α*_*m*_ as parameter *z*, with *ε*_*r*_ and *σ* as x and y, respectively, for each MUT sample. Taking as a reference the permittivity value obtained in subsection 3.1 and averaging the respective indices of *α*_*m*_ and *a*_*m*_ of *θ*_*i*_ measured, it is possible to obtain the conductivity *σ* by determining the modified transmission coefficient *α*_*m*_ that is closest to the *a*_*m*_ index, according to minimization of the r.m.s. error using the expression given in Eq (12), similarly to that used in [19].

$$\begin{array}{c} e_{r.m.s}=\sqrt{{\left(\frac{1}{N}\sum_{\theta_{i}}\left|a_{m}(\theta_{i})-\alpha_{m}(\theta_{i})\right|\right)}^{2}(dB)}\\ for\;\theta_{i}=0{}^{\circ},15{}^{\circ},30{}^{\circ}\;and\;45{}^{\circ}.\\ N=4. \end{array}$$ (12)

**Fig 11:**
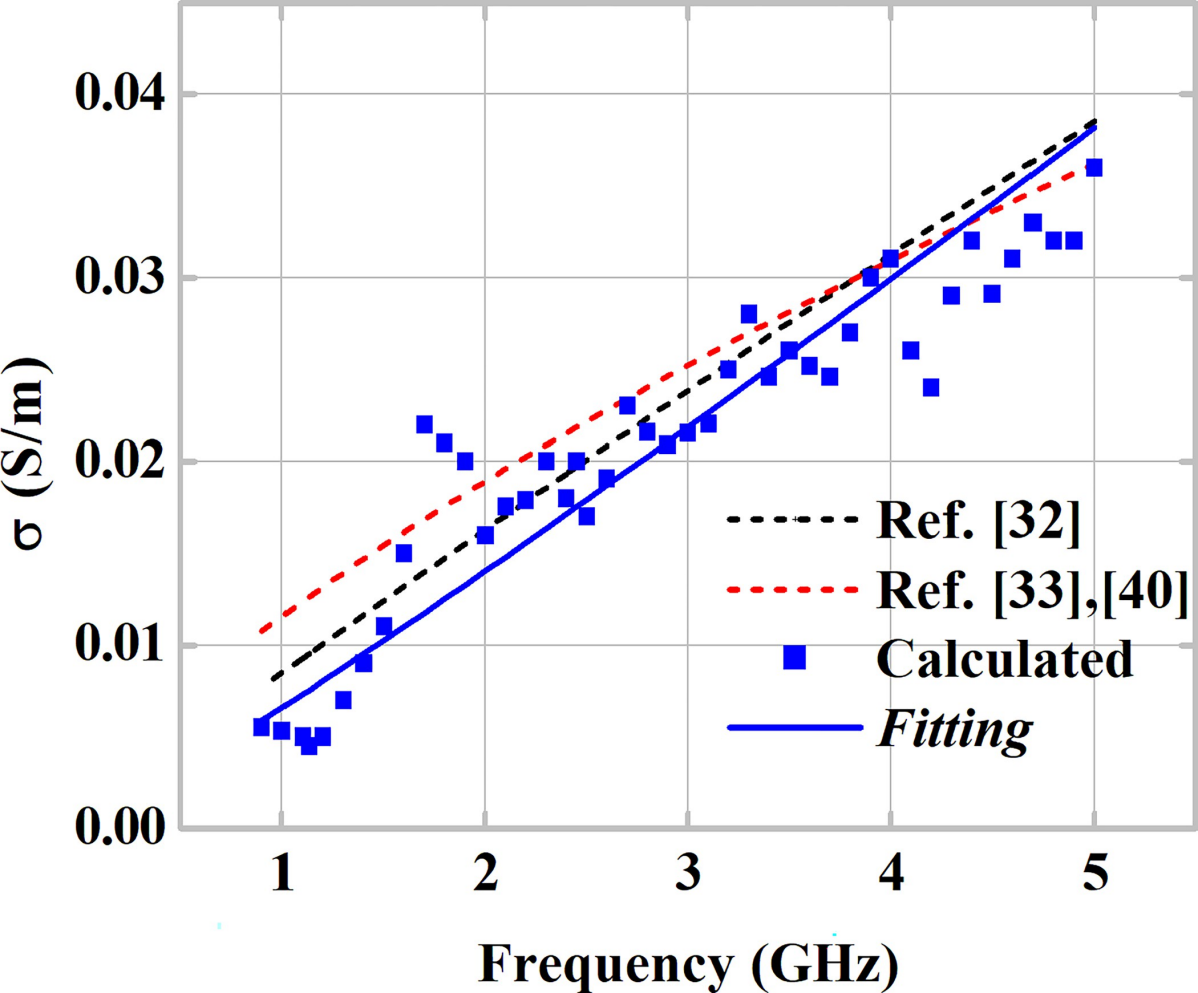
Conductivity obtained via Ray Tracing method as a function of frequency. Comparison with curves available in the literature.

Once the coefficients that minimize the error function (12) are defined for the averages of the measurements at different values of *θ*_*i*_, the attenuation indices are obtained for each analyzed frequency. With these indices, conductivity values are calculated for each frequency using the previously established matrices. Fig 11 presents the calculated results of conductivity *σ* as a function of the operating frequency, which represent the arithmetic mean of the three MUT samples. The results are compared with those of the curves reported in references [37, 38, 40]. In Fig 11, the presented results and their fitting demonstrate an increase in the conductivity of the gypsum block samples with increasing frequency. Comparison between the reference lines and the line obtained for conductivity demonstrates consistency within the study frequency range.

## 4. Conclusion

The non-invasive electrical characterization of plaster blocks used in the construction of walls is presented in this work. Measured and calculated values of the electrical parameters of Shielding Effectiveness (SE), complex relative permittivity, loss tangent, attenuation coefficient and conductivity are obtained for a wide range of frequencies in which several traditional and new wireless communication standards are considered. In an innovative way, two classic methodologies are combined, the first called the NRW Method is used to determine the characteristics of electrical permittivity, and the second, known as the Ray Tracing Method, is used to obtain conductivity values for the analyzed plaster blocks. Different from the classic methods for determining dielectrial properties that a prior estimation of the data occurs to enable sampling parameters to be obtained, this work presented an innovative way of obtaining them entirely based on the analysis of the material through frequency measurements, which results in a greater level of precision of the data results. This work presented an efficient method for determining the dielectric properties of walls that are fundamental for the design of indoor wireless communications links for modern wireless and mobile communications systems, including 5G and IoT. The scheme of using both techniques results in the proposal of a new methodology for the characterization of solid materials, being applied here in the determination of the electrical parameters of plaster blocks used in the construction of walls. The data calculated and obtained through an extensive campaign of measurements prove the effectiveness of the proposed methodology. The electrical parameters characterized for the plaster blocks demonstrate an accurate level when compared to values described in the related literature. Therefore, the presented study appears as a new alternative in the process of characterization of solid materials used in civil construction, and can be used as a reference for applications involving the study of the RF channel in built environments.
